# The diversification of the basic leucine zipper family in eukaryotes correlates with the evolution of multicellularity

**DOI:** 10.1186/s12862-016-0598-z

**Published:** 2016-02-01

**Authors:** Katia Jindrich, Bernard M. Degnan

**Affiliations:** School of Biological Sciences, The University of Queensland, Brisbane, QLD 4072 Australia

**Keywords:** bZIP transcription factor, Gene regulatory networks, Evolution, Complexity

## Abstract

**Background:**

Multicellularity evolved multiple times in eukaryotes. In all cases, this required an elaboration of the regulatory mechanisms controlling gene expression. Amongst the conserved eukaryotic transcription factor families, the basic leucine zipper (bZIP) superfamily is one of the most ancient and best characterised. This gene family plays a diversity of roles in the specification, differentiation and maintenance of cell types in plants and animals. bZIPs are also involved in stress responses and the regulation of cell proliferation in fungi, amoebozoans and heterokonts.

**Results:**

Using 49 sequenced genomes from across the Eukaryota, we demonstrate that the bZIP superfamily has evolved from a single ancestral eukaryotic gene and undergone multiple independent expansions. bZIP family diversification is largely restricted to multicellular lineages, consistent with bZIPs contributing to the complex regulatory networks underlying differential and cell type-specific gene expression in these lineages. Analyses focused on the Metazoa suggest an elaborate bZIP network was in place in the most recent shared ancestor of all extant animals that was comprised of 11 of the 12 previously recognized families present in modern taxa. In addition this analysis identifies three bZIP families that appear to have been lost in mammals. Thus the ancestral metazoan and eumetazoan bZIP repertoire consists of 12 and 16 bZIPs, respectively. These diversified from 7 founder genes present in the holozoan ancestor.

**Conclusions:**

Our results reveal the ancestral opisthokont, holozoan and metazoan bZIP repertoire and provide insights into the progressive expansion and divergence of bZIPs in the five main eukaryotic kingdoms, suggesting that the early diversification of bZIPs in multiple eukaryotic lineages was a prerequisite for the evolution of complex multicellular organisms.

**Electronic supplementary material:**

The online version of this article (doi:10.1186/s12862-016-0598-z) contains supplementary material, which is available to authorized users.

## Background

Increasing evidence suggests that the evolution of complex multicellular organisms arose from the expansion and diversification of gene regulatory networks (reviewed in [1]). In eukaryotes, the precise control of gene expression, often in response to physiological and environmental stimuli, largely depends on the binding of specific transcription factor proteins to specific DNA sequences [2]. This ancient mode of gene regulation has been co-opted into and is instrumental in the ontogeny of multicellular eukaryotes, sitting at nodes in developmental gene regulatory networks (GRNs) that underlie spatiotemporal and cell type-specific gene transcription [3]. Analysis of GRNs, largely in bilaterian animals, reveals they are populated by transcription factors of differing evolutionary age, with most being either unique to metazoans (e.g. nuclear receptors) or of an older evolutionary origin (e.g. basic helix-loop-helix transcription factors) [4].

The basic leucine zipper (bZIP) superfamily of transcription factors appears to have originated early in eukaryotic evolution [2]. bZIPs sit at the heart of key pathways regulating cellular decisions across this domain of life [5]. They have been consistently implicated in a wide range of core eukaryotic cellular processes, including cell proliferation and differentiation, stress response and homeostasis [6]. However, the ancestral role of bZIPs in eukaryotes has been difficult to infer because a single conserved function has not been identified amongst living eukaryotes.

bZIP transcription factors take their name from a highly conserved 60–80 amino acid bZIP domain, which has a bipartite organisation consisting of an N-terminal basic region, responsible for DNA binding, and a leucine zipper, which mediates homo- and hetero-dimerization between bZIPs. As dimers, they regulate transcription by binding short DNA target sites, often in the form of 8 base pair palindromes. In recent years, the bZIP network of several eukaryotes has been described, and their evolution has been, to some extent, investigated in animals [7, 8], fungi [9] and plants [10]. The recent sequencing of disparate eukaryotic genomes now allows the search for the primordial bZIP and the reconstruction of the evolutionary trajectories this family has taken in different higher eukaryotic lineages, including phyla, kingdoms and superkingdoms.

Here, we analysed the bZIP gene repertoires from a wide range of eukaryote genomes, focusing on the lineage with the widest coverage of draft genomes, the holozoans. We traced back metazoan bZIP families to their origin in the holozoan last common ancestor, opisthokont last common ancestor and beyond. By comparing bZIPs diversification in the main eukaryotic clades, we demonstrate that bZIPs originated from a single protein and then evolved independently in each major eukaryotic lineage. bZIP family complexity appears to increase incrementally over long evolutionary periods, prior to evolutionary transitions into a complex multicellular condition. Early in eukaryotic evolution, a first expansion phase occurred independently in each of the four main eukaryotic lineages - Opistokonta, Amoebozoa, Planta and Heterokonta- yielding to 3 or 4 bZIP families. These families constitute the core of the bZIP complement in extant fungi, heterokonts and amoebozoans. A second expansion phase occurred in holozoans and early plants, prior to the emergence of complex multicellularity in either lineage. In holozoans, it occurred in two main steps: one before the divergence of unicellular holozoans, which display numerous examples of colonial forms with life cycles comprised of more than one cell type (e.g. in *Salpingoeca rosetta* [11] and *Capsaspora owczarzaki* [12]), and one during the early evolution of metazoans, prior to the emergence of the crown group.

## Results

### Accrual of the animal bZIP repertoire over the course of holozoan evolution

**Fig. 1:**
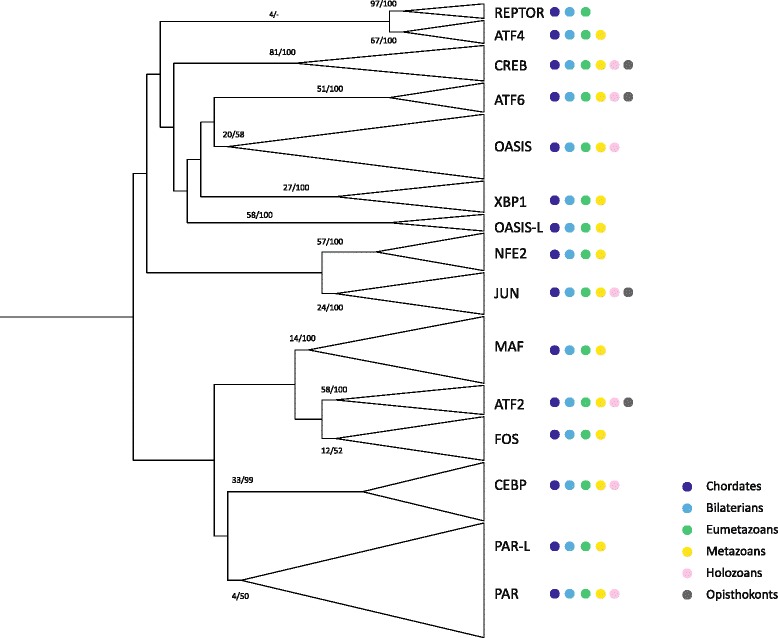
Phylogenetic analysis of the metazoan bZIP superfamily. Mid-point rooted maximum likelihood tree of unambiguously identified bZIPs. Branches are collapsed into families. Statistical support was obtained with 1000 bootstrap replicates and Bayesian posterior probabilities; both values are shown on key branches. Coloured circles to the right indicate the presence of members of that family in taxa listed to the right

446 bZIP genes were identified from 18 metazoan (nine bilaterian, three cnidarian, one placozoan, two ctenophore, three sponge) and two unicellular holozoan (a choanoflagellate and a filasterean) genomes, using an iterative process that included (i) screening predicted proteomes using PFAM, (ii) interrogating the coding sequences in the genome by blastP and (iii) then using hidden Markov models (HMMs) constructed using the bZIP sequences identified in each clade to re-interrogate each of the proteomes (Additional file 1: Table S1). By comparing and undertaking phylogenetic analyses of the bZIPs from each holozoan against three reference sets of bZIPs (bZIPs identified in *Homo*, *Branchiostoma* and poriferans, which is a composite of the bZIPs found in the genomes of a representative demosponge, calcisponge and homoscleromorph), we identified 12 bZIP families that were comprised of at least one human orthologue, and one family (REPTOR) and two sub-families (PAR-Like and OASIS-Like) that appear to have been lost in humans (Fig. 1, Additional files 2, 3 and 4 and Additional file 1: Table S2).

Phylogenetic analyses suggest that PAR-L and OASIS-L are related to PAR and OASIS (Additional file 2 and 3). PAR-L bZIPs possess the two Asn residues at positions 13 and 14, characteristic of the PAR and CEPB families (Additional file 2). OASIS-L bZIPs include the OASIS-specific Tyr at position 27 but exhibit a unique combination of amino acids at positions 13 to 21: NAIxAxxNR (x represents variable residues), instead of the typical NKxSAxxSR OASIS combination (Additional file 2). REPTOR however does not appear to be related to any other bZIP family and is characterised by an astonishingly conserved basic domain, with 33 consecutive residues (positions 7–35, 37–39 and 41) identical in nearly all species, and a very short coiled-coil region (Fig. 1 and Additional file 2). We named this family after its *Drosophila* orthologue, which has been recently identified as a downstream factor of the TORC1 signalling pathway [13]. The origin of the PAR-L and OASIS-L sub-families, and REPTOR family can be traced back to being present in the last common metazoan and eumetazoan ancestor, respectively.

**Fig. 2:**
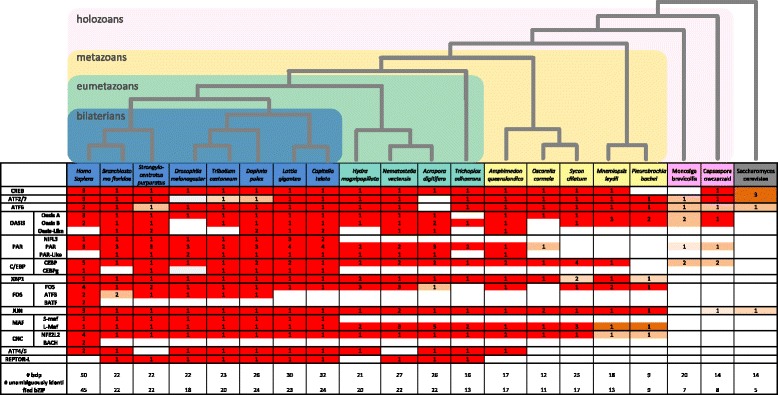
Evolution of recognized bilaterian bZIP families. Listed in the first column are bZIP families (left) and subfamilies (right). Along the top are the species included in this survey and their phylogenetic relationship based on [14, 57]. The number of genes per bZIP family and subfamily are listed below each species. Colours reflect the level of evidence behind this tally (decreasing from red to orange). For each species the total number of bZIPs identified and the number of bZIPs retrieved are reported at the bottom of the table

Nearly all bZIP families and subfamilies have been maintained in metazoan taxa included in this survey, although there are few cases of gene loss. Notably, ctenophores, which are deemed to be the earliest branching metazoan phyletic lineage [14], exhibit a higher level of bZIP gene loss than any other metazoan lineage (Fig. 2 and Additional file 1: Table S2). As a number of bZIP genes missing in the ctenophores surveyed are present in non-metazoan holozoans – PAR, CREB and CEBP in *Pleurobrachia* and PAR in *Mnemioposis* - the ctenophore bZIP repertoire is not likely to reflect the ancestral metazoan condition. We conclude that at least eleven of bilaterian orthology groups were present in the last common ancestor to extant metazoans, and seven in the holozoan LCA.

Notably, this approach identified a putative member of the JUN family in the holozoan *Capsaspora*, a bZIP gene family previously regarded as metazoan-specific [7]. This finding is consistent with our observation that members of the fungal GCN4 family and the metazoan JUN family appear to be distant orthologues (Fig. 4 and Additional file 1: Table S2).

**Fig. 3:**
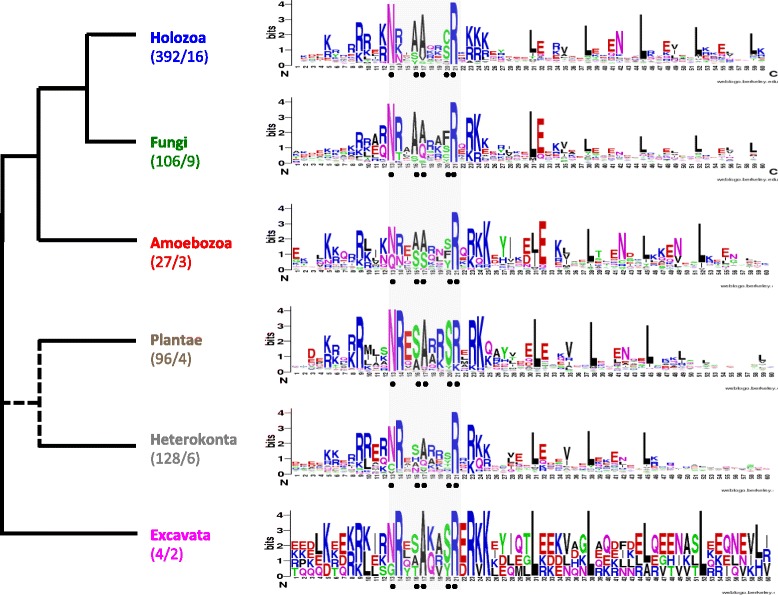
Conservation and evolution of bZIPs in eukaryotes. The consensus bZIP domain for each of the six main eukaryotic lineages is depicted with a logo [58]. The number of sequences/species used in this analysis is indicated in parentheses. Black circles indicate five highly conserved residues of the basic domain throughout eukaryote, which have been shown to be involved in DNA-binding site recognition in animals and yeast [17]

Thus, our approach identified (i) the foundational bZIP family present prior to the evolution of metazoan multicellularity, (ii) the origin of metazoan bZIP families (OASIS A-B, MAF-NFE2) and bilaterian subfamilies (sMAF, NFIL3, CEBPg, ATF3), (iii) the expansion and loss of specific family members in particular lineages, and (iv) uncovered three uncharacterised bZIP families (Figs. 2 and 3 and Additional files 2 and 3).

### Emergence of novel domain combinations in holozoan bZIP families

bZIPs appear to have increased their connectivity throughout metazoan evolution through the linking to other protein domains [6]. The kinase inducible activation domain (KID) is associated with CREB in several metazoans and mediates the interaction of CREB and p300/CBP. Although the *Capsaspora* genome encodes p300 [8], its *CREB* gene does not encode a KID. In contrast both sponge and ctenophore CREBs include a KID domain, consistent with a domain-shuffling event early in metazoan evolution to bring together these domains.

A detailed analysis of domains associated with bZIP domains in other families identified a number of conserved domain combinations, including an ATF2-specific and a JUN-specific transactivation domain, previously described in human ATF2 [15] and JUN [16], and an ATF6-specific domain, which is present in unicellular holozoan orthologues (Additional file 5). We also recovered highly conserved regions located N-terminally of the PAR, MAF and OASIS basic domain, the latter being present in unicellular holozoan OASISes (Additional file 5). Interestingly, these regions were not conserved in the PAR-L and OASIS-L bZIPs. None of these domains were detected in fungal orthologues or related bZIPs.

### Conservation of eukaryotic bZIP DNA-binding motif

Based on our observation that many bilaterian and indeed metazoan bZIPs originated early in holozoan evolution, well prior to the diversification of the Metazoa, we attempted to retrace bZIP evolution beyond the opisthokont last common ancestor. A total of 896 bZIPs were identified from 49 eukaryotic genomes, including opisthokont (metazoans, choanoflagellates, and fungi), amoebozoan, plant (Viridiplantae - land plants and green algae, and red algae), heterokont (oomycetes, diatoms and brown algae) and excavate representatives. bZIP genes were recovered in all species surveyed (Additional file 1: Table S1), except two amoebozoans (*Hartmannella vermiformis and Physarum polycephalum*) and a microsporidial fungus (*Antonospora locustae*), consistent with bZIPs being present in the most recent shared ancestor of extant eukaryotes.

Amongst the eukaryotic bZIPs, the leucine residues in the leucine zipper region (residues 31, 38, 45, 52 and 59 in Fig. 3) and a nine amino acid region in the N-terminal of the basic DNA-binding domain (residues 13–21 in Fig. 3) are the most conserved. Five very highly conserved amino acids within the basic region - Asn13, Ala16, Ala17, Ser/Cys20 and Arg21 - have been shown to be instrumental in determining sequence-specific DNA binding in bilaterians and fungi [17, 18]. The first and last of these amino acids are the most conserved amongst eukaryotes, with only three differences at these positions found across all eukaryotes surveyed: the excavate *Giardia* has a solitary bZIP factor with a Gly at position 13; a few plants have Lys at position 21; and in oomycete heterokonts have a Cys at position 13 in several proteins that appear to be functional [18]. The level of conservation of the other three residues varies between eukaryotic lineages (Fig. 3). For instance, nonpolar Ala is the most common residue in position 16 in opisthokonts and ameobozoans, while in plants, heterokonts and excavates this site is most often populated by Ser, which has an uncharged polar side chain (Fig. 3). Amongst the most conserved residues, position 20 is the most variable in terms of residue diversity and disparity, with Cys only observed in this position in opisthokonts.

### Independent expansion of bZIPs in different eukaryotic lineages

**Fig. 4:**
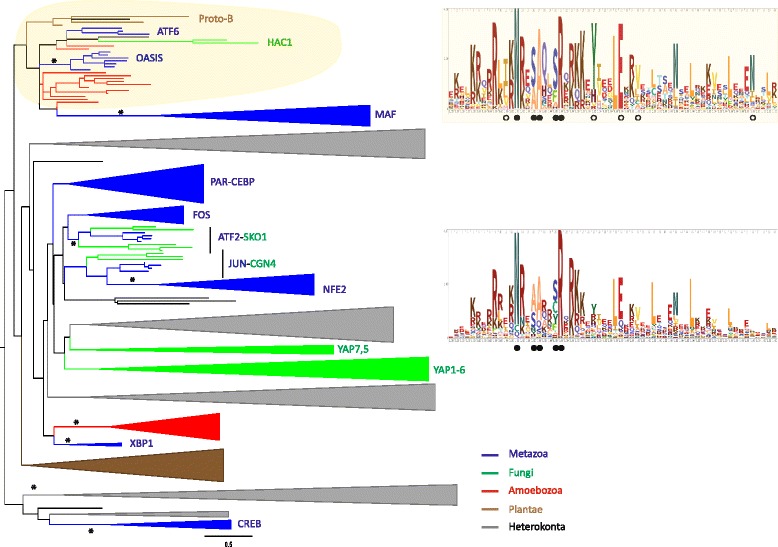
Phylogenetic analysis of eukaryotic bZIPs. Unrooted Maximum Likelihood tree of bZIPs from two species representing each of the five main eukaryotic lineages: metazoans (blue); fungi (green); amoebozoans (red); plants (brown); and heterokonts (black). To avoid crowding the tree, the *Arabidopsis* bZIP set was restricted to two genes from each bZIP family (as described in [10]). Branches are collapsed into kingdom-specific clades and the name is indicated when possible. Statistical support was obtained with 1000 bootstrap replicates and an asterisk indicates well-supported branches (>50 %). A unique cluster containing orthologues from the metazoan OASIS-ATF6 family, the fungal HAC1 family and the plant group proto-B group is highlighted in yellow. bZIPs from this clade share five additional conserved positions, indicated by white circles (top logo, yellow background), compared to the whole eukaryotic set (bottom logo). Black circles indicate five highly conserved positions of the bZIP domain discussed earlier

As eukaryotic bZIPs appear to have originated from a single ancestral protein [2], we sought to identify any orthologues present in extant representatives of the six main lineages (Fig. 3). Given the large number of sequences, each analysis included only one or two representative species from each of these lineages, although we ran multiple permutations, changing the lineage representatives each time. In general, the topology of the resulting trees remained constant regardless of the representative used. As with previous studies that included bZIPs from different eukaryotic groups [7, 8, 19], the alignments do not contain enough informative positions for meaningful bootstrap analysis, which impedes the inference of phylogenetic relationships. Nonetheless, we consistently recovered one cluster, which includes bZIPs from metazoans (OASIS-ATF6 family), fungi (HAC1 family), plants (proto-B group) and heterokonts (Fig. 4), suggesting an ancestral bZIP existed prior to the divergence of these eukaryotic lineages. Interestingly, those families are among the founder bZIP families of each kingdom. This grouping is further supported by the high conservation of five unique residues (Fig. 4).

**Fig. 5:**
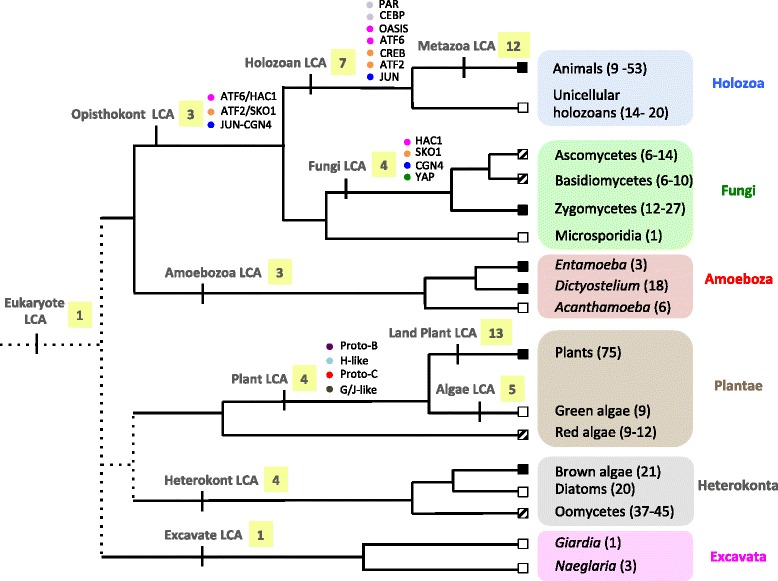
A model for the eukaryotic bZIP evolution. A simplified phylogenetic tree of the eukaryotes is depicted. Phylogenetic relationships are based on the most commonly accepted view of the phylogeny of the Eukaryotes [59]. A white square indicates multicellular lineages; a black square indicates unicellular lineages; a black and white square indicate lineages comprising both multicellular and unicellular species. In parentheses, we reported the range of bZIPs we identified in the species sampled in this study. The estimated number of bZIPs in each last common ancestor (LCA) is indicated in a yellow box next to its name. When possible, we indicate the inferred bZIP families by a circle. Two circles have the same colour when they are thought to have originated from the same protein

In most cases, bZIP family expansions are restricted to individual organismal lineages (Fig. 4), with orthologues only identifiable within a given eukaryotic lineage (Fig. 5). The bZIP superfamily has been divided into orthology groups in both metazoans [5] and plants [10]. These usually coincide with DNA-binding and dimerization preferences [17, 20]. Consistent with previous studies [5, 9], we identified 13 bZIP families in metazoans and five in fungi. We also recovered 13 clusters in plants, which correspond to the 13 families described in [10], and five in the green alga *Chlamydomonas*. In heterokonts, both maximum likelihood and Bayesian analyses support four ancestral orthologue groups. The limited number of genomes available in Amoebozoa and the debatable phylogenetic relationship between amoebozoan species greatly limit our analysis. We tentatively identified 3 groups of orthologues in this kingdom.

## Discussion

Using 49 sequenced genomes representing disparate eukaryotic lineages, including 18 representative metazoan genomes, we have reconstructed the evolution of one of the most ancient transcription factor families employed in animal development and disease, the bZIPs. We demonstrate that the evolution of multicellularity is correlated with the expansion and diversification of bZIPs in different families. However, an apparent increase in morphological and behavioural complexity (e.g. along the bilaterian or eumetazoan stem) is not always accompanied with an increase in gene family number. Indeed, bZIP family complexity appears to increase incrementally over long evolutionary periods, probably being one of a number of prerequisites for the evolution of networks underlying complex gene regulation.

### The metazoan bZIP network is comprised of members of differing age

**Fig. 6:**
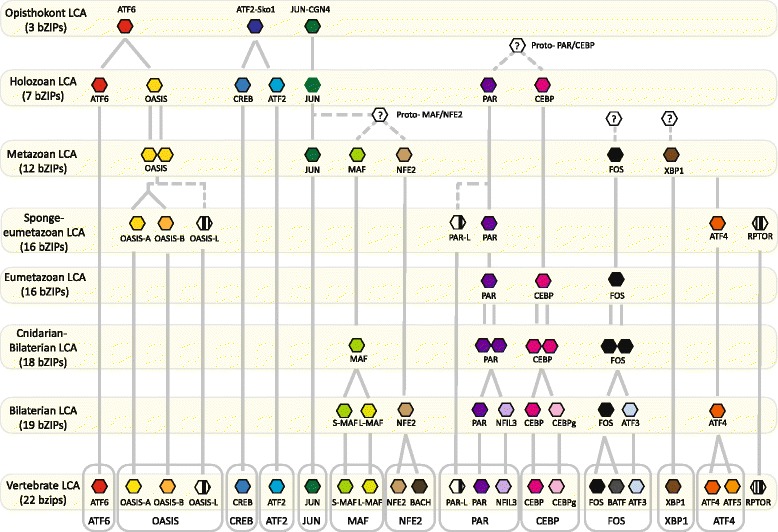
Origin and diversification of holozoan bZIPs. Each row represents the putative bZIP complement present in the corresponding LCA (last common ancestor). An hexagon represents a bZIP protein, and the family it belongs to is indicated underneath. Main families are indicated by different colours while subfamilies are indicated with different shades. bZIP subfamilies that had not been reported prior to this study are marked with stripes. Vertical lines represent families or subfamilies that have been conserved throughout metazoan evolution. A double line indicates a duplication event; a fork indicates the divergence of one of the copies. A dotted line reflects an uncertain relationship. In the case of CEBP-PAR and MAF-NFE2, we ascertained that the two subfamilies originated from a single protein but could not identify this ancestor (depicted as an hexagon bearing a question mark). At the bottom, subfamilies are gathered into the traditional bilaterian main families

The phylogenetic analysis of metazoan bZIPs highlights three major periods in the evolution of bZIPs (Fig. 6). The first diversification of the metazoan bZIP complement occurred prior to the divergence of holozoan lineages. There were at least three identifiable ancestral opisthokont bZIPs families, ATF6, ATF2-sko1 and Jun-CGN4, that expanded into 7 holozoan families. A second round of expansion and diversification occurred prior to the divergence of extant metazoan lineages, with all of the 13 metazoan bZIP families evolving prior the divergence of eumetazoan and sponge lineages. Based on the bZIPs present in early branching metazoans, we infer that the last common ancestor to all animals possessed minimally 12 bZIPs (Fig. 6). This complement has remained remarkably stable over the course of metazoan evolution, with very little evidence of gene loss. Four bZIP families (MAF, PAR, CEBP and FOS) duplicated and underwent further diversification prior to bilaterian cladogenesis; three families underwent another round of duplication in stem chordates (NFE2) and vertebrates (ATF4 and FOS) (Fig. 6).

The OASIS family of bZIPs form three orthology groups (Additional file 3) that emerged either prior to metazoan cladogenesis or before the divergence of sponge and eumetazoan lineages. The OASIS-L group is distinct from the other OASIS family members and lacks the OASIS-specific extended basic domain. PAR, CEBP and FOS duplicated and diverged along the bilaterian stem to give rise to three new pairs of families, PAR-NFIL3, CEBP-CEBPg and FOS-ATF3. Cnidarians also possess pairs of CEBP and FOS genes that clade with one of the bilaterian orthologues, suggesting that these duplicated prior to the divergence of cnidarians and bilaterians; one of the duplicates subsequently diverged to form a new subfamily in bilaterians. The sponge-eumetazoan common ancestor possessed two PAR orthologues: PAR and PAR-L; PAR further duplicated and diversified in stem bilaterians to give rise to PAR and NFIL3 orthology groups. Independent expansion of this family in bilaterians, cnidarians and poriferans has yielded PAR-members that are unique to these taxa (Additional file 3). Similarly, although all eumetazoans possess at least two MAF-bZIPs, the emergence of large MAF and small MAF orthology groups did not occur until after the divergence of cnidarians and bilaterians (Additional file 3). This study also identified a new bZIP family, called REPTOR after its *Drosophila* member [13], which emerged from an unidentified bZIP ancestor along the poriferan-eumetazoan stem. All REPTORs display an almost identical basic region and a very short leucine zipper, which may nonetheless be functional for dimerization [13, 21].

ATF2-Sko1, ATF6-HAC1 and JUN-CGN4 families are common between metazoans and fungi, and most likely reflect the bZIP network that was in place in the common ancestor of extant opisthokonts. Members of those families preferentially recognise a palindromic sequence that is conserved in extant animals and fungi [6]. We found that the transactivation of ATF2, CREB and JUN are conserved throughout metazoans, and that of ATF6 throughout holozoans (Additional file 5). The conservation of both co-factors and binding sites potentially reflects the primordial role of these factors in the opisthokont ancestor and points towards a conserved function across animal and fungal orthologues. Consistent with this idea, metazoan ATF6 and yeast HAC1 hold a similar role in the activation of the seemingly conserved unfolded protein response [22]. Similarly, ATF2 plays a key role in the control of homeostasis in animals (reviewed in [23]) while SKO1 is central in the yeast response to different stress stimuli [24]. This reasoning may explain the general housekeeping role of CREB/ATF factors in animals, in contrast to the specific roles of metazoan-specific families (e.g. PAR and the control of circadian rhythm [25]).

### The diversification of bZIPs in eukaryotes support a role in the evolution of multicellularity

Multicellularity evolved several times in eukaryotes [26] and arose from a diversification of gene regulatory networks [1]. Given the bZIPs are part of the ancestral eukaryotic transcription factor repertoire, a relationship between bZIP expansion, and diversification and evolution of complex multicellularity appears plausible. Our analysis supports the inference that the eukaryotic LCA possessed a solitary bZIP, which underwent an early expansion and diversification in the lineages leading to crown animals and fungi (stem opisthokont lineage), and Viridiplantae (land plants and green algae) (Fig. 5). This is consistent with an early increase in bZIP membership being a prerequisite for the evolution of the complex multicellularity displayed in animals, plants and fungi. The bZIP superfamily underwent a further duplication and divergence in each of these lineages, as detailed above for animals, with some of the expansions occurring prior to the emergence of the stem metazoan lineage (Figs. 5 and 6).

The differential expansion of the bZIP superfamily in opisthokont and plant lineages is consistent with the hypothesis that the independent diversification of bZIP families contributed to evolution of complex multicellularity on more than one occasion. Specifically a marked difference in ancestral metazoan and fungal, and land plant and algal bZIP repertoires, with morphologically more complex metazoans and land plants having more bZIP families (12 and 13, respectively) than simpler fungi and unicellular algae (4 and 5, respectively; Fig. 5).

The bZIP superfamily has also diversified in other eukaryotic lineages that display colonial or simple multicellularity. For instance, with a more limited dataset we identified 3 and 4 families in amoebozoans and heterokonts, respectively. In these cases again it appears that the expansion and diversification of bZIPs into different families occurs in lineages that include organisms with complex life cycles and more than one cell type (Fig. 5). The establishment of the core of the bZIP network early in evolution is consistent with bZIPs central role in basic cellular processes [5].

### Reconstruction of the ancestral bZIP

The giardial bZIP has been proposed as a model for the precursor of all bZIPs [7]. However, it is consistently recovered in a clade that lacks most eukaryotic lineages; only metazoan and fungal bZIPs clade with this bZIP. Although in this study we could not confidently identify the ancestral bZIP of each kingdom, the similarity between sequences from ancient families of plant (proto-B), metazoan (ATF6-OASIS), fungal (HAC1), amoebozoan and heterokont bZIPs suggests they share features of the ancestral eukaryotic bZIP (Fig. 3), including the deeply conserved NxxSAxxSR (residues 13–21) signature motif.

This five-residue motif is involved in sequence-specific DNA binding and has not varied greatly over the entire course of the bZIP superfamily evolution. Indeed this may explain the restricted number bZIP families and the similarity of bZIP DNA-binding sequences throughout Eukaryota. Although each monomer possesses its own transactivation activity (reviewed in [27]), bZIPs can nonetheless regulate a wide range of cellular processes because they bind DNA as dimers, where each basic domain contributes individually to the recognition of one half binding site [17]. Pairing of bZIPs generates an extensive array of dimeric regulators, which in combination determines the transcriptional activity. Thus the independent diversification of bZIPs in multiple eukaryotic lineages allows for a marked and lineage-specific expansion in potential combinations. As dimerization is key to the functional diversification of bZIP transcription factors, offering the potential for flexible and complex transcriptional programs, the expansion of this gene family potentially contributed to the foundations underlying the evolution of complex multicellular organisms.

## Conclusions

We compiled a dataset of 896 bZIPs from 49 sequenced genomes from across the Eukaryota. The depth of this dataset permits an assessment of the evolution of this family of transcription factors in relation to the timing of the major evolutionary transitions in eukaryotes, including the evolution of multicellularity. We demonstrated that bZIPs underwent an early expansion and diversification, independently in each of the five main eukaryotic lineages, and was likely a contributing prerequisite for the evolution of organisms with complex life cycles and multiple cell types. Focusing on metazoans, we reconstructed the duplication events that shaped bZIP sub-families and identified three previously uncharacterised bZIP sub-families that appear to have been lost in mammals. Our analysis identified the ancestral metazoan and holozan bZIP repertoire, which comprise 7 and 12 founder genes, respectively.

## Methods

### Taxonomic sampling and retrieval of bZIPs

The sequences of the full complement of bZIP genes were retrieved from the fully sequenced genomes of 31 non-metazoan eukaryotes and 18 metazoan representatives. Metazoan opisthokonts include: *Homo sapiens, Branchiostoma floridae and Ciona intestinalis* (Chordata); *Strongylocentrotus purpuratus* (Echinodermata); *Drosophila melanogaster, Tribolium castaneum* and *Daphnia pulex* (Arthropoda): *Lottia gigantea* (Mollusca)*, Capitella telata* (Polychaeta); *Nematostella vectensis, Acropora digitifera* and *Hydra magnipapillata* (Cnidaria); *Trichoplax adherans* (Placozoa)*; Mnemiopsis leydi* and *Pleurobrachia bachei* (Ctenophora); and *Amphimedon queenslandica, Oscarella carmela* and *Sycon ciliatum* (Porifera). Non-metazoan opisthokonts include: *Monosiga brevicollis* (Choanoflagellata)*; Capsaspora owczarzaki* (Filasterea); *and Saccharomyces cerevisiae, Schizosaccharomyces pombe, Candida albicans, Aspergillus nidulans, Magnaporthe grisea, Ustilago maydis, Cryptococcus neoformans, Mucor circinelloides, Rhizophagus irregularis*, *Antonospora locustae* and *Encephalitozoon cuniculi* (Fungi). Plants include*: Arabidopsis thaliana* (Planta)*; Chlamydomonas reinhardtii* (green alga)*;* and *Galdieria sulphuraria* and *Chondrus Crispus* (red alga). Amoebozoans include: *Entamoeba histolica, Dictyostelium discoideum, Acanthamoeba castellanii, Hartmannella vermiformis and Physarum polycephalum.* Heterokonts include*: Thalassiosira pseudonana, Phytophthora sojae, Pythium ultimum, Phytophthora infectans, Ectocarpus sliculosus* (brown alga)*, Hyaloperonospora arabidopsidis,* and *Phaeodactylum tricornutum;* Excavates include*: Giardia lamblia and Naeglaria gruberi*. As there are few published analyses of heterokont bZIPs, we sampled several species in this lineage. Subsequent phylogenetic analyses in this study were restricted to *Phytophthora infectans* (oocmycete), *Thalassiosira pseudonana* (diatom) and *Ectocarpus siliculosus* (brown alga). Species and data sources information can be found in Additional file 1: Table S1. All databases were publically available and no animal work requiring ethics approval was conducted.

The complete bZIP set for each species was obtained through an iterative process including (i) building an initial set of bZIP proteins using PFAM, (ii) interrogating the proteome by BlastP in several databases (NCBI, JGI, Ensemble and species specific genome browsers [28–42]) and then (iii) re-interrogating each of the proteomes with a HMMER genome-wide scanning using custom Hidden Markov Models generated for each phylogenetic clade. When searching early branching holozoans, additional models were generated for each phylum. A general cutoff value of 10^-4^ was used but proteins with higher e-value were also manually selected against the identification criteria listed below. When possible, we interrogated our dataset with previously published data to assess completeness (see Additional file 1: Table S1 for details, [7, 9, 10, 18, 20, 21, 43–45]). Putative bZIPs were then manually inspected for the following features: (1) a basic domain BR, as defined by [20], and (2) a leucine zipper, within the two heptads located C-terminally of the BR and presenting a coil-coiled structure of two heptads minimum.

### Phylogenetic analyses

We defined the N-terminal domain boundary of the bZIP domain as the N-terminal end of the crystal structure of GCN4 in complex with DNA [46] and to avoid artefacts in the tree building algorithm, we set the length of the basic domain (basic region and leucine zipper) at 60 amino acids. Protein sequences were trimmed to their bZIP domain and aligned using the MAFFT v7 algorithm [47] in Geneious and then manually inspected. Maximum likelihood (ML) analyses were carried out by RaxML [48]. The LG + G substitution model was scored as the best-fit model for each alignment, using ProtTest [49]. Branch support was estimated by performing 1000 bootstrap replicates. Bayesian analyses were carried out with MrBayes 3.2 [50], using the LGG substitution matrix, with 2 parallel runs, four chains and a resampling frequency of 100. Different temperatures were used when convergence was not achieved. We considered that we had reached convergence when the average standard deviation of split frequencies fell below 0.05. The analysis was terminated if convergence was not reached after 12,000,000 generations.

To determine orthologue assignments, we looked at each species independently.

For holozoan bZIPs, we constructed three bZIP reference sets including the sequences from *Homo*, *Branchiostoma* and poriferans (bZIPs from a representative demosponge, calcisponge and homoscleromorph), respectively. We made multiple alignments comprising the putative bZIPs of each species and one of the reference set; the bZIPs identified in each phylum; and the bZIPs identified in each clade. Phylogenetic trees were inferred by maximum likelihood and Bayesian analyses.

We considered two sequences as orthologues if their grouping was supported by bootstrap values and posterior marginal probabilities superior to 80 % and 90 %, respectively. We then manually inspected each protein for conserved amino acids peculiar to each bZIP family (described in [51] (CEBP); [52] (MAF); [17] (JUN and FOS); [53] (PAR); [54] (CREB) and [55] (all) and Additional file 4). Protein sequences, names and family assignment can be found in Additional file 1: Table S1.

Using a similar method, we sought to identify groups of orthologues within and between six eukaryotic kingdoms: animals, fungi, plants, amoebozans, heterokonts and excavates. Our classification was compared with previous studies that have focused on a specific clade (in fungi [9] and plants [10]), to confirm our orthologue assignments and limited to taxa for which there is an available draft genome. Thus the eukaryotic lineages with wider and deeper coverage are likely to be more accurate. To reduce the effect of potentially unstable sequences, we ran multiple permutations of the same analysis, with only one or two species representative of each kingdom or lineage, changing the lineage representatives each time.

### Search for other motifs and domains in bZIP-containing proteins

The complete set of full-length bZIP sequences from the following representative holozoan species was searched for the presence of conserved motifs using the MEME suite [56]: *Homo sapiens*; *Drosophila melanogaster; Hydra magnipapillata; Amphimedon queenslandica; Mnemiopsis leydii; Monosiga brevicollis;* and *Capsaspora owczarzaki*. The minimal and maximal width for a motif was set to 6 and 50 residues, respectively. The motifs found to be conserved between orthologues were investigated further by building HMM for these motifs/domains and searching the entire derived bZIP proteome of all holozoan species.

### Availability of supporting data

The data sets supporting the results of this article are included within the article and its additional files. Protein sequences can be found in Additional file 1: Table S1. Additional alignments and trees are available upon request.

## Additional files

Additional file 1:
**Table S1.** bZIP dataset. First tab contains databases information, method of protein retrieval and references. Following tabs include the full list of the protein sequences used in this study, accession information and, when relevant, family assignment. **Table S2.** Statistical support for family assignment. We reported the boostrap values (NJ: neighbor-joining and ML: maximum likelihood) and posterior probabilities (BI: Bayesian inference) that supported the assignment of each protein to a bZIP family, listed on the left. Taxa are listed at the top. (ZIP 151 kb) Additional file 2:
**Phylogenetic analysis of the bZIP complement in Metazoa.** (A) Mid-point rooted maximum likelihood tree of unambiguously identified bZIPs from 6 representative species (3 bilaterians (*H. sapiens*, *B. floridae* and *D. pulex*), 1 sponge (*A. queenslandica*), 1 filasterean (*C. owczarzaki*) and 1 fungus (*S. cerevisiae*). Poriferan branches are shown in orange, filasterean branches in pink and fungal branches in green. bZIP families are shaded in grey. (B) Alignments of PAR-L, OASIS-L and REPTOR subfamilies. Shading indicates residue conservation at a given position, decreasing from black (100 %) to light grey (60 %). Typical PAR- and OASIS- bZIPs are included at the top of the alignment for reference; triangles indicate the positions discussed in the text. (PDF 1491 kb) Additional file 3:
**Diversification of bZIP subfamilies.** Mid-point rooted Bayesian inference trees of the bZIP members of five families: OASIS, PAR, FOS-ATF3, CEBP and MAF. Posterior probabilities are displayed on the branches. *Hsap: Homo Sapiens;* Brfl*: Branchiostoma floridae;* Spur: *Strongylocentrotus purpuratus*; Dmeg: *Drosophila melanogaster*; Tcas: *Tribolium castaneum*; Dapu: *Daphnia pulex*; Lotg: *Lottia gigantean;* Ctel: *Capitella telata*; Nmev: *Nematostella vectensis;* Hmag*: Hydra magnipapillata;* Adi: *Acropora digitifera*;Tadh*: Trichoplax adherans;* Amq*: Amphimedon queenslandica;* Mnle*:* Mnemiopsis leidyi. (PDF 81 kb) Additional file 4:
**Family-specific amino acid analysis.** Based on sequence similarity, we built a consensus sequence for each family. The number of sequences used to build this consensus is indicated in parentheses. As a reference, the canonical bZIP sequence is shown in the box above the alignment and black circles indicate five highly conserved residues of the basic domain discussed in the text. We only display residues that are specific to a bZIP family; residues that are conserved in all bZIPs appear as a dot in the alignment. Yellow highlighted residues are positions that are most specific to each family and that were used to confirm family assignment in this study. (PDF 932 kb) Additional file 5:
**Emergence of new domain combinations in metazoan bZIP families.** A bar represents a typical bZIP family member, on which we indicated the positions of the bZIP domain (BRLZ, in yellow) and a conserved motif (in grey). Three of these motifs – KID in CREB, TAD in ATF2 and HOB 1-2 in JUN- have been previously described in bilaterian bZIPs. Each motif is displayed as a logo. Coloured circles to the right indicate the presence of this motif in the taxa listed on the top right. *Hs: Homo sapiens; Dm: Drosophila melanogaster; Hm: Hydra magnipapillata; Aq: Amphimedon queenslandica; Mb: Monosiga brevicollis; Co: Capsaspora owczarzaki; Sc: Saccharomyces cerevisiae*. (PDF 1097 kb)
