# Supplementary material for: The diversification of the basic leucine zipper family in eukaryotes correlates with the evolution of multicellularity
Source: BMC Evol Biol. 2016 Feb 1;16:28. doi: 10.1186/s12862-016-0598-z (PMC4736632; doi:10.1186/s12862-016-0598-z)
Supplement: Additional file 2: — Phylogenetic analysis of the bZIP complement in Metazoa. (A) Mid-point rooted maximum likelihood tree of unambiguously identified bZIPs from 6 representative species (3 bilaterians (H. sapiens, B. floridae and D. pulex), 1 sponge (A. queenslandica), 1 filasterean (C. owczarzaki) and 1 fungus (S. cerevisiae). Poriferan branches are shown in orange, filasterean branches in pink and fungal branches in green. bZIP families are shaded in grey. (B) Alignments of PAR-L, OASIS-L and REPTOR subfamilies. Shading indicates residue conservation at a given position, decreasing from black (100 %) to light grey (60 %). Typical PAR- and OASIS- bZIPs are included at the top of the alignment for reference; triangles indicate the positions discussed in the text. (PDF 1491 kb) [file 12862_2016_598_MOESM2_ESM.pdf]

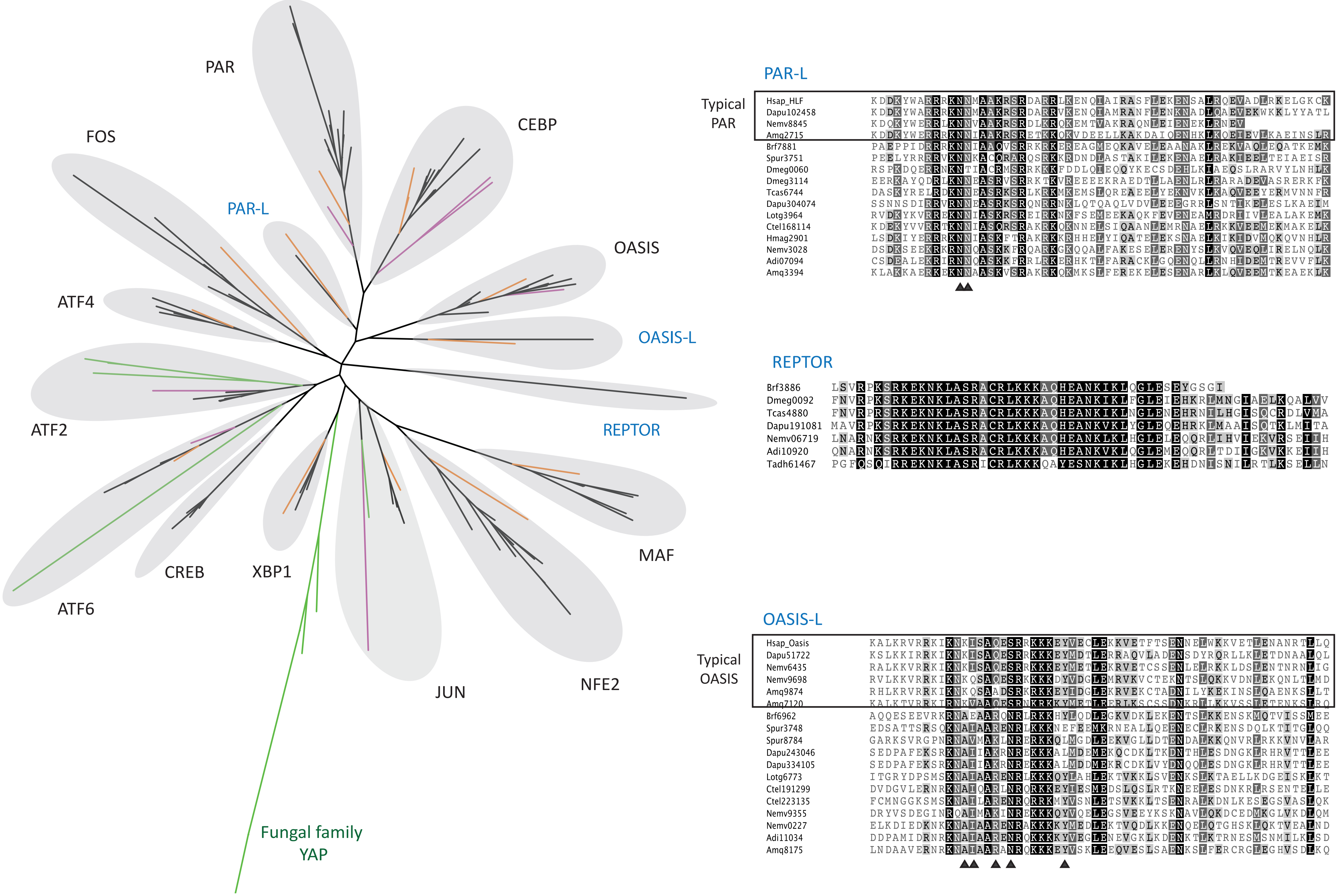
PAR
CEBP
FOS
PAR-L
OASIS
ATF4
OASIS-L
ATF2
REPTOR
ATF6
CREB
XBP1
MAF
JUN
NFE2
Fungal family
YAP
PAR-L
Typical PAR
Hsap_HLF KDDKYWARRRKNNMAAKRSRDARRLKENQIAIRASFLEKENSALRQEVADLRKELGKCK
Dapu102458 KDDKYWARRRKNNMAAKRSRDARRVKENQIAMRANFLENKNADLQAEVEKWKKLYYATL
Nemv8845 KDQKYWERRLKNNVAAKRSRDLKRQKEMTVAKRAQNLEIENEKLRNEV
Amq2715 KDDKYWERRRKNNIAAKRSRETKKQKVDEELLKAKDAIQENHKLKQEIEVLKAEINSLR
Brf7881 PAEPPIDRRRKNNIAAQVSRRKRKEREAGMEQKAVELEAANAKLREKVAQLEQATKEMK
Spur3751 PEELYRRRRVKNNKACQRARQSRKKRDNDLASTAKILEKENAELRAKIEELTEIAEISR
Dmeg0060 RSPKDQERRNKNTIACRMSRRKKKFDDLQIEQQYKECSDEHLKIAEQSLRARVYLNHLK
Dmeg3114 EERKAYQDRLKNNEASRVSRRKTKVREEEEKRAEDTLLAENLRLRARADEVASRERKFK
Tcas6744 DASKYRELRDKNNEASRKSRLKRKMKEMSLQREAEELYEKNVKLKAQVEEYERMVNNFR
Dapu304074 SSNNSDIRRVRNNEASRKSRQNRRNKLQTQAQLVDVLEEEGRRLSNTIKELESLKAEIM
Lotg3964 RVDKYKVRREKNNIASKRSREIRKNKFSEMEEKAQKFEVENEAMRDRIIVLEALAKEMK
Ctel168114 KDEKYVVRRTKNNIASQRSRAKRKQKNNELSIQAANLEMRNAELRKKVEEMEKMAKELK
Hmag2901 LSDKIYERRRKNNIASKFTRAKRKKRHHELYIQATELEKSNAELKIKIDVMQKQVNHLR
Nemv3028 DSDKSEEKRKRNNQASKKFRQARKGKQQALFAKESELERENYSLKVQVEQLIRELNQLK
Adi07094 CSDEALEKRIRNNQASKKFRRLRKERHKTLFARACKLGQENQLLRNHIDEMTREVVFLK
Amq3394 KLAKKAERKEKNNAASKVSRAKRKQKMKSLFEREKELESENARLKLQVEEMTKEAEKLK
REPTOR
Brf3886 LSVRPKSRKEKNKLASRACRLKKKAQHEANKIKLQGLESEYGSGI
Dmeg0092 FNVRPKSRKEKNKLASRACRLKKKAQHEANKIKLFGLEIEHKRLMNGIAELKQALVV
Tcas4880 FNVRPRSRKEKNKLASRACRLKKKAQHEANKIKLNGLENEHRNILHGISQCRDLVMA
Dapu191081 MAVRPKSRKEKNKLASRACRLKKKAQHEANKVKLYGLEQEHRKLMAAISQTKLMITA
Nemv06719 LNARNKSRKEKNKLASRACRLKKKAQHEANKLKLHGLELEQQRLIHVIEKVRSEIIH
Adi10920 QNARNKSRKEKNKLASRACRLKKKAQHEANKVKLQGLEMEQQRLTDIIGKVKKEIIH
Tadh61467 PGFQSQIRREKNKIASRICRLKKKQAYESNKIKLHGLEKEHDNISNILRTLKSELLN
OASIS-L
Typical OASIS
Hsap_Oasis KALKRVRRKIKNKISAQESRRKKKEYVECLEKKVETFTSENNELWKKVETLENANRTLLQ
Dapu51722 KSLKKIRRKIKNKISAQESRRKKKEYMDTLERRAQVLADENSDYRQRLLKLETDNAALQL
Nemv6435 RALKKVRRKIKNKISAQESRRKKKEYMETLEKRVETCSSENLELRKKLDSLENTNRNLIG
Nemv9698 RVLKKVRRKIKNKQSAQESRKKKKDYVDGLEMRVKVCTEKNTSLQKKVDNLEKQNLTLMD
Amq9874 RHLKRVRRKIKNKQSAADSRKRKKEYIDGLEKRVEKCTADNILYKEKINSLQAENKSLLT
Amq7120 KALKTVRRKIRNKVAAQESRNKRKKYMETLEERLKSCSSDNKRLLKKVSSLETENKSLRQ
Brf6962 AQQESEEVRKRNAEAARQNRLRKKHYLQDLEGKVDKLEKENTSLKKENSKMQTVISSMEE
Spur3748 EDSATTSRSQKNAIAARENRLKKKNEFEEMKRNEALLQEENECLRKENSDQLKTITGLQQ
Spur8784 GARKSVRGPNRNAVMAKLNRERKKQLMGDLEEKVGLLDTENDALKKQNVRLRKKVNVLAR
Dapu243046 SEDPAFEKSRKNAIIAKRNREKKKALMDEMEKQCDKLTKDNTHLESDNGKLRHRVTTLEE
Dapu334105 SEDPAFEKSRKNAIIAKRNREKKKALMDDMEKRCDKLVYDNQQLESDNGKLRHRVTTLEE
Lotg6773 ITGRYDPSMSKNAIAARENRLKKKQYLAHLEKTVKKLSVENKSLKTAELLKDGEISKLKT
Ctel191299 DVDGVLERNRKNAIQARLNRQRKKEYIESMEDSLQSLRTKNEELESDNKRLRSENTELLE
Ctel223135 FCMNGGKSMSKNAILARENRQRRKMYVSNLETSVKKLTSENRALKDNLKESEGSVASLQK
Nemv9355 DRYVSDEGINRQAIMAKINREKKKQYVQELEGSVEEYKSKNAVLQKDCEDMKGLVKDLQM
Nemv0227 ELKDIEDKNKKNAIAARENRAKKKKYMEDLEKTVQDLKKENQELQTGHSKLQKTVEALND
Adi11034 DDPAMIDRNRKNAIAARENRQKKKKYVEGLENEVGKLKDENKTLKTRNESMSNMILKLSD
Amq8175 LNDAAVERNRKNAIAARANRQKKKEYVSKLEEQVESLSAENKSLFERCRGLEGHVSDLKQ
