# Supplementary figures and images for: The diversification of the basic leucine zipper family in eukaryotes correlates with the evolution of multicellularity
Source: BMC Evol Biol. 2016 Feb 1;16:28. doi: 10.1186/s12862-016-0598-z (PMC4736632; doi:10.1186/s12862-016-0598-z)

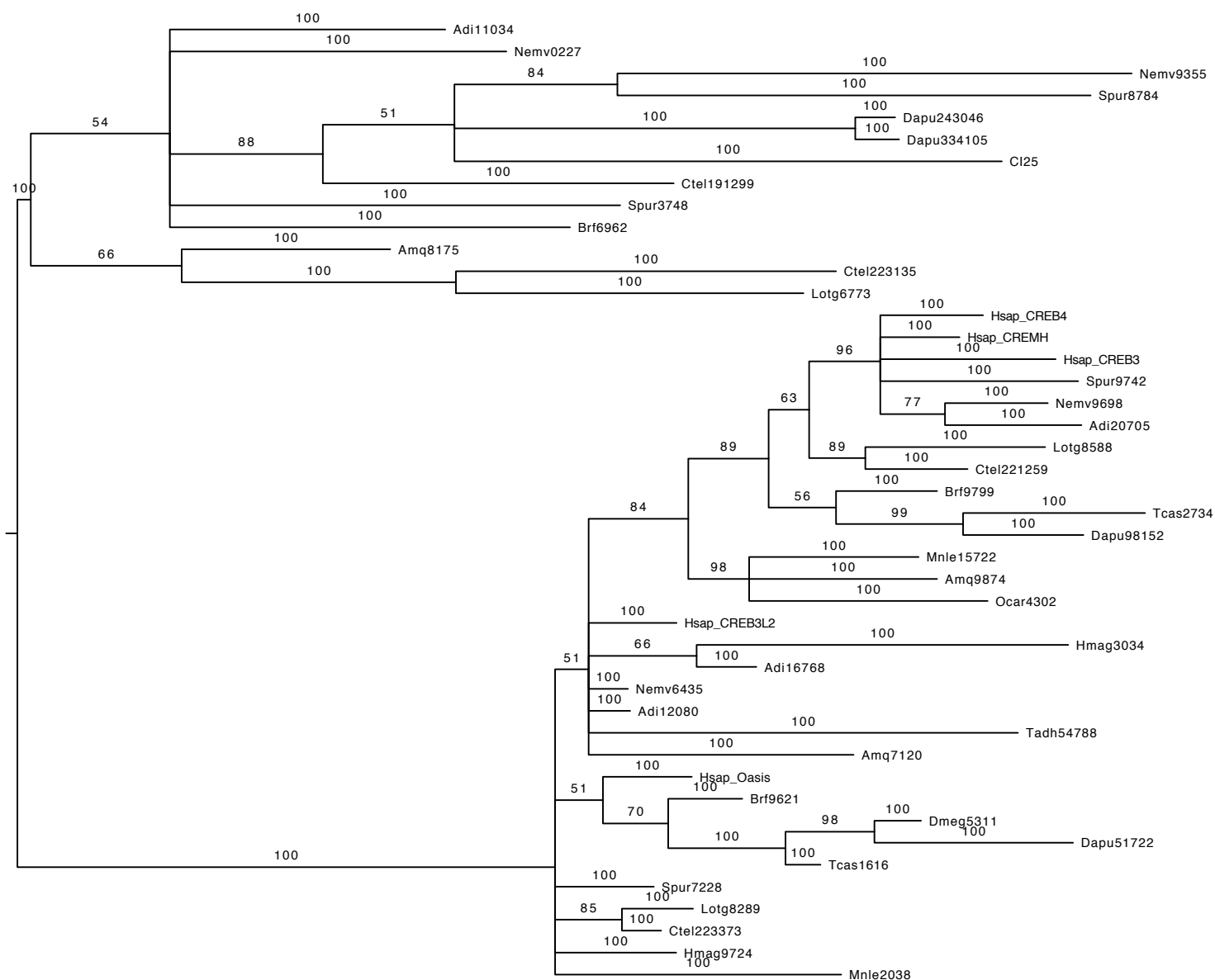

0.2

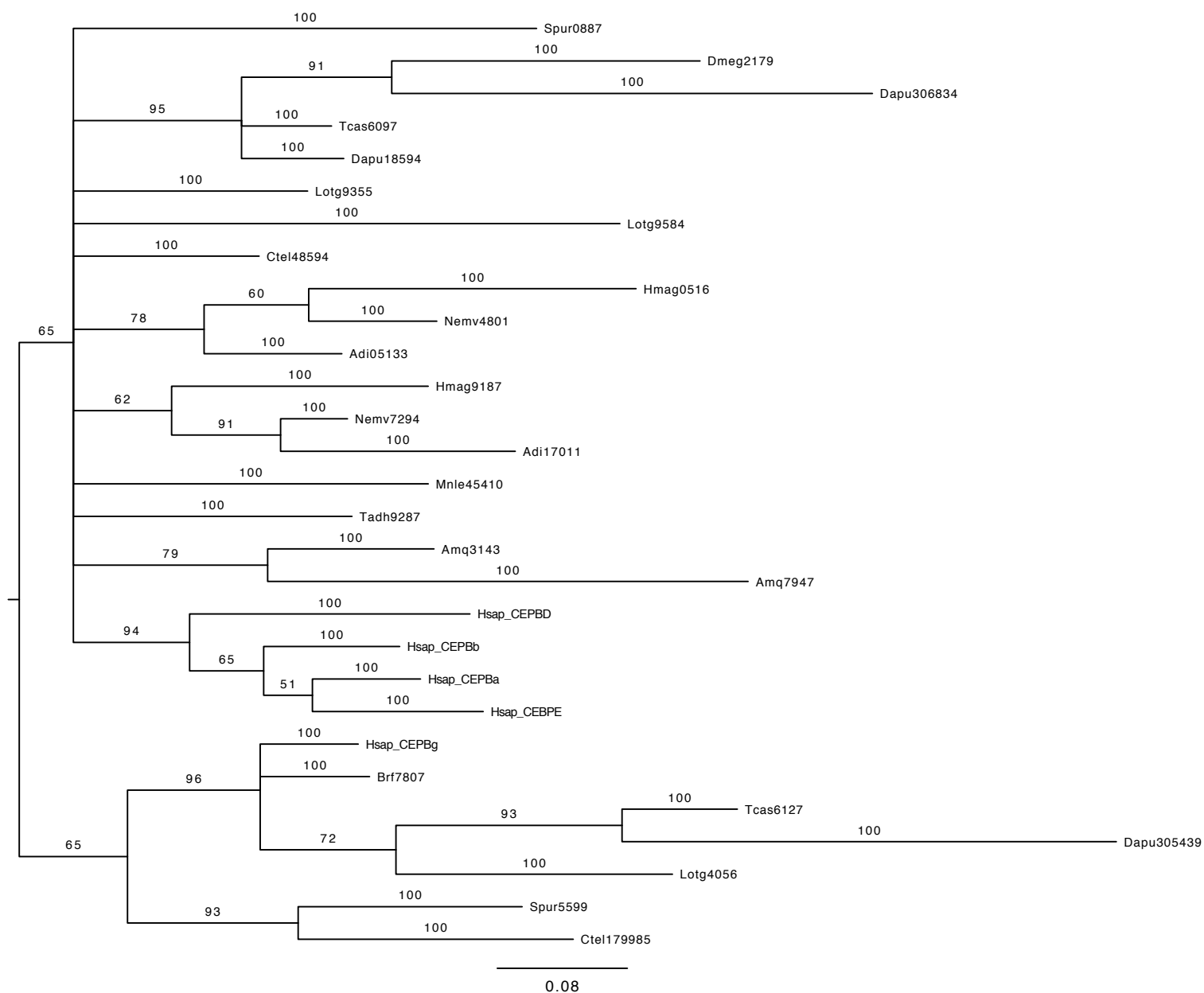

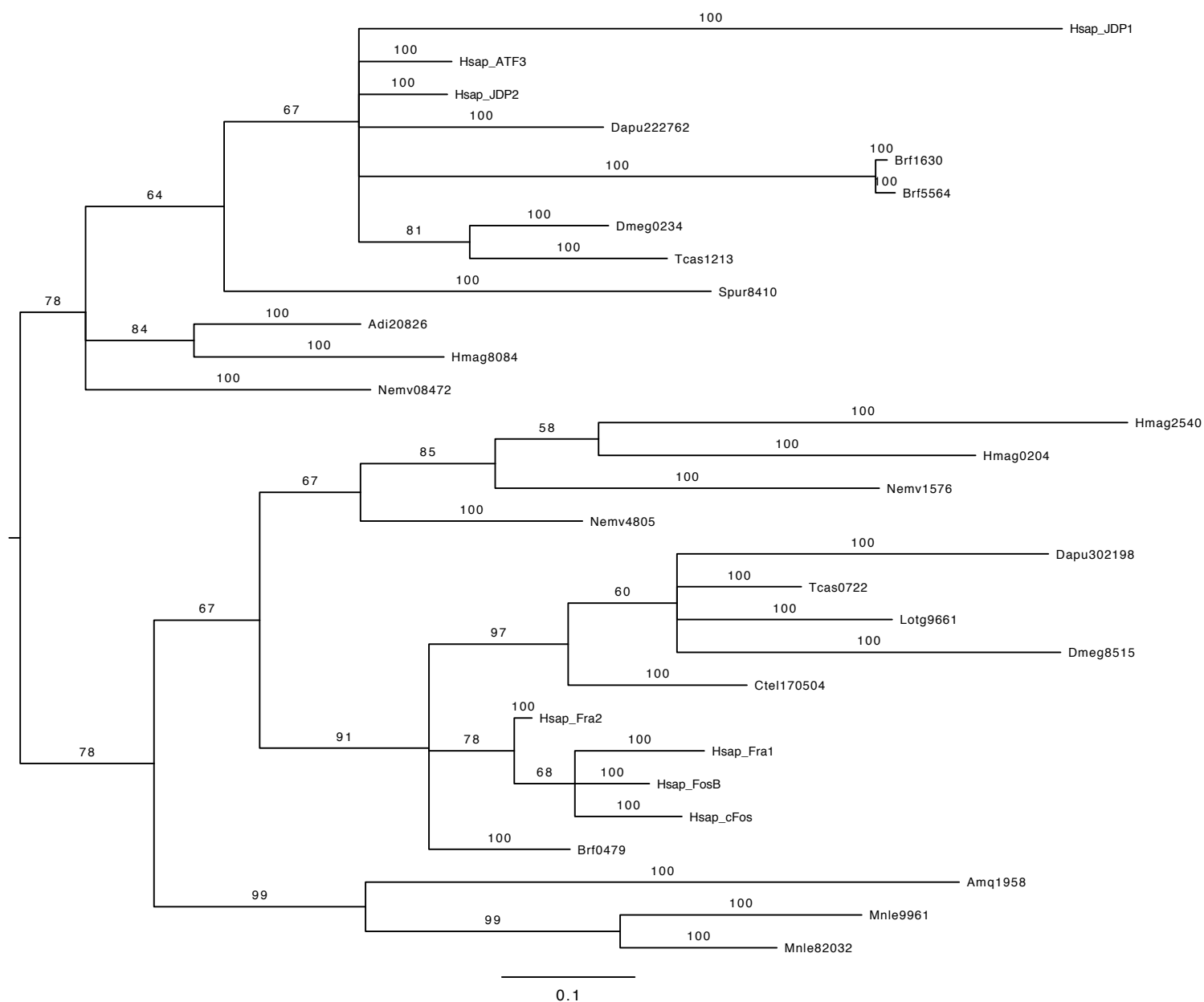

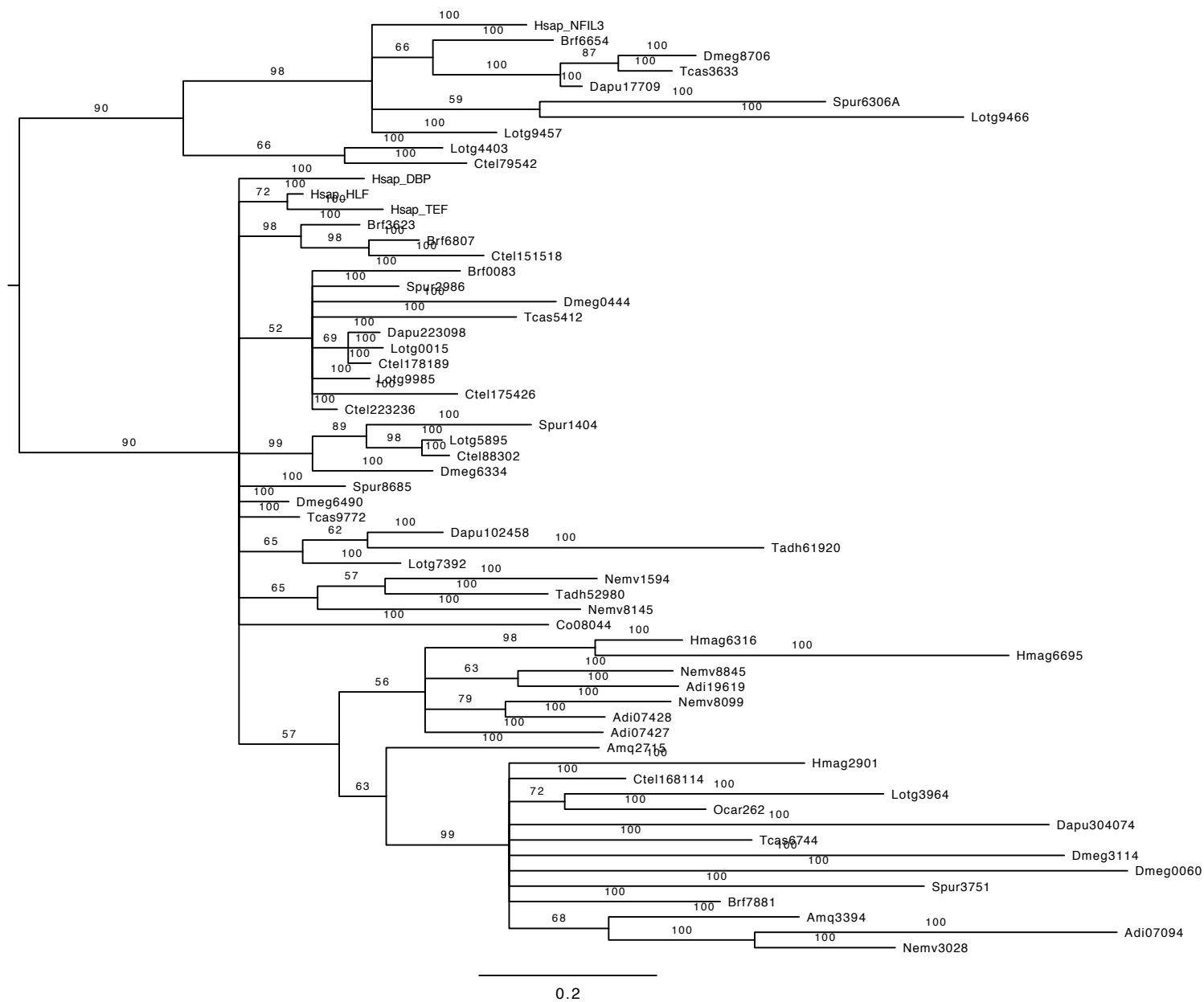

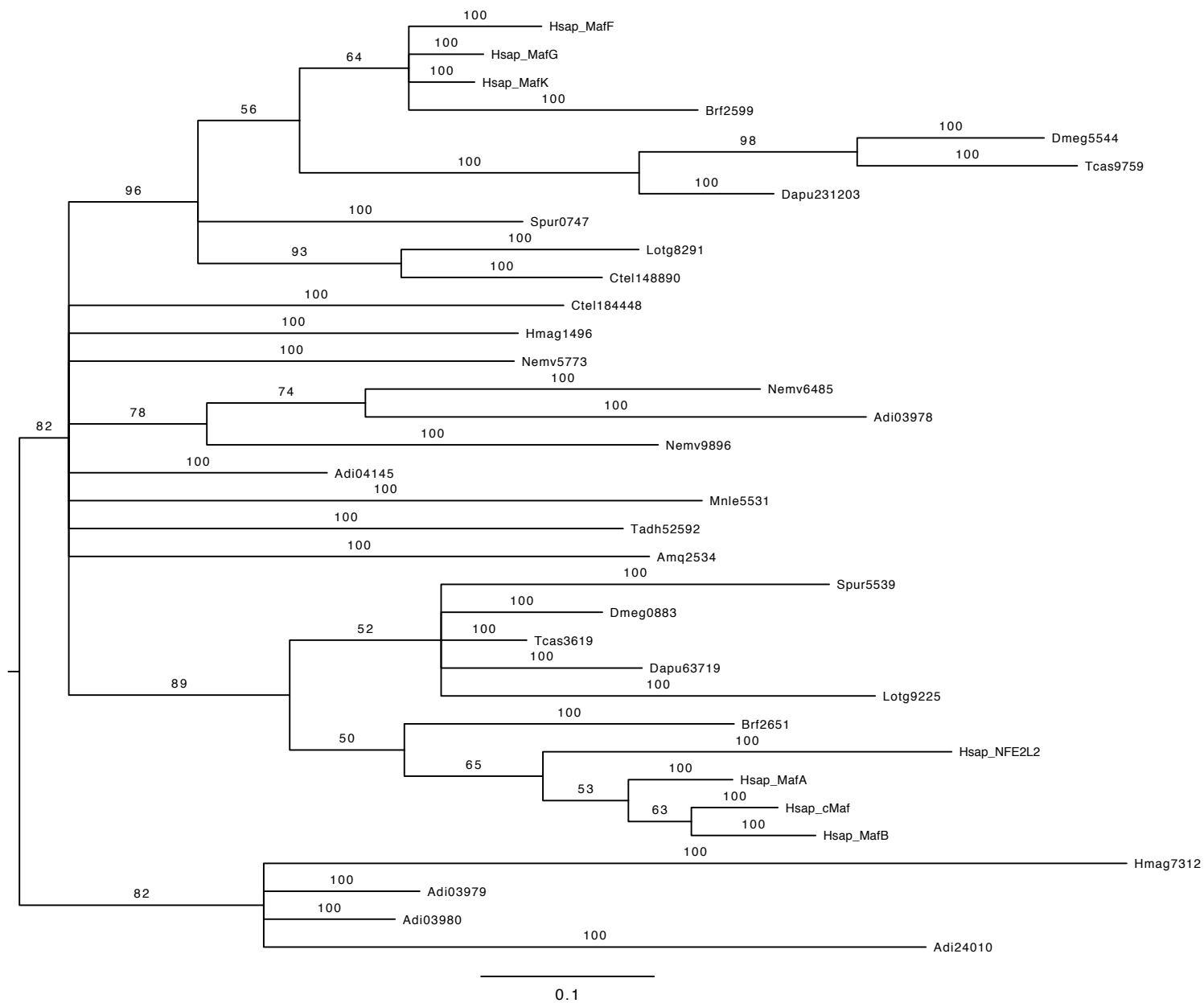

Supplement: Additional file 3: — Diversification of bZIP subfamilies. Mid-point rooted Bayesian inference trees of the bZIP members of five families: OASIS, PAR, FOS-ATF3, CEBP and MAF. Posterior probabilities are displayed on the branches. Hsap: Homo Sapiens; Brfl: Branchiostoma floridae; Spur: Strongylocentrotus purpuratus; Dmeg: Drosophila melanogaster; Tcas: Tribolium castaneum; Dapu: Daphnia pulex; Lotg: Lottia gigantean; Ctel: Capitella telata; Nmev: Nematostella vectensis; Hmag: Hydra magnipapillata; Adi: Acropora digitifera;Tadh: Trichoplax adherans; Amq: Amphimedon queenslandica; Mnle: Mnemiopsis leidyi. (PDF 81 kb) [file 12862_2016_598_MOESM3_ESM.pdf]

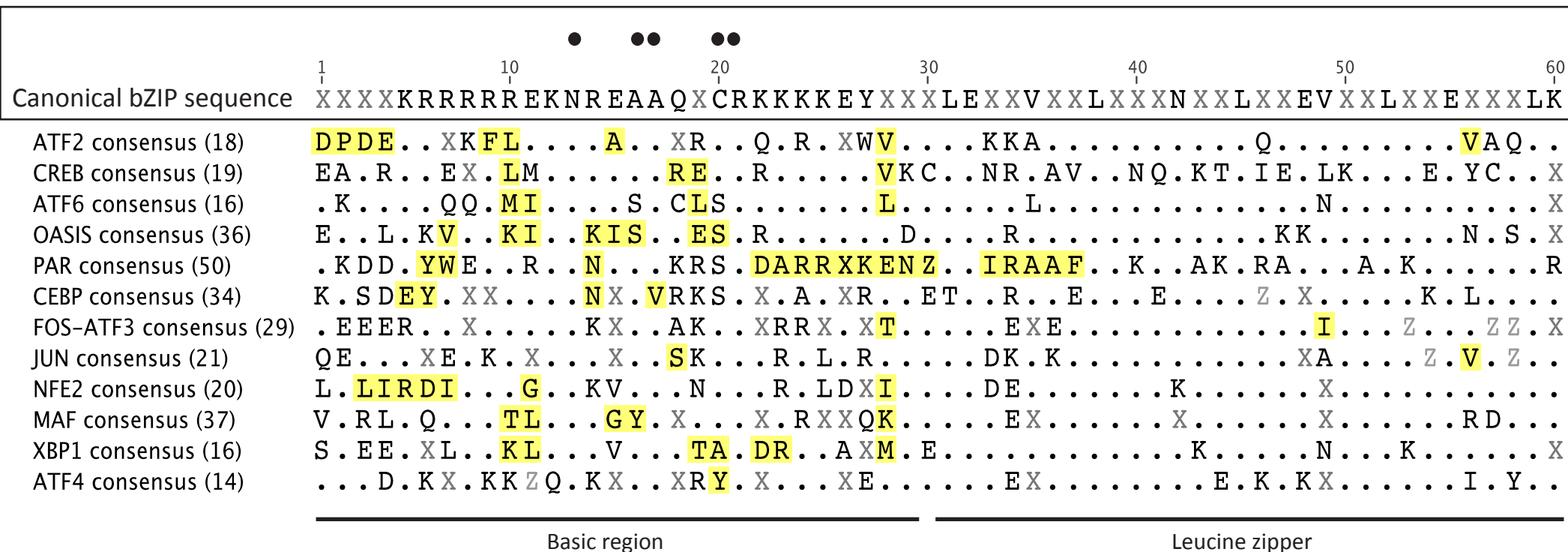

Supplement: Additional file 4: — Family-specific amino acid analysis. Based on sequence similarity, we built a consensus sequence for each family. The number of sequences used to build this consensus is indicated in parentheses. As a reference, the canonical bZIP sequence is shown in the box above the alignment and black circles indicate five highly conserved residues of the basic domain discussed in the text. We only display residues that are specific to a bZIP family; residues that are conserved in all bZIPs appear as a dot in the alignment. Yellow highlighted residues are positions that are most specific to each family and that were used to confirm family assignment in this study. (PDF 932 kb) [file 12862_2016_598_MOESM4_ESM.pdf]
