# Supplementary material for: The diversification of the basic leucine zipper family in eukaryotes correlates with the evolution of multicellularity
Source: BMC Evol Biol. 2016 Feb 1;16:28. doi: 10.1186/s12862-016-0598-z (PMC4736632; doi:10.1186/s12862-016-0598-z)
Supplement: Additional file 5: — Emergence of new domain combinations in metazoan bZIP families. A bar represents a typical bZIP family member, on which we indicated the positions of the bZIP domain (BRLZ, in yellow) and a conserved motif (in grey). Three of these motifs – KID in CREB, TAD in ATF2 and HOB 1-2 in JUN- have been previously described in bilaterian bZIPs. Each motif is displayed as a logo. Coloured circles to the right indicate the presence of this motif in the taxa listed on the top right. Hs: Homo sapiens; Dm: Drosophila melanogaster; Hm: Hydra magnipapillata; Aq: Amphimedon queenslandica; Mb: Monosiga brevicollis; Co: Capsaspora owczarzaki; Sc: Saccharomyces cerevisiae. (PDF 1097 kb) [file 12862_2016_598_MOESM5_ESM.pdf]

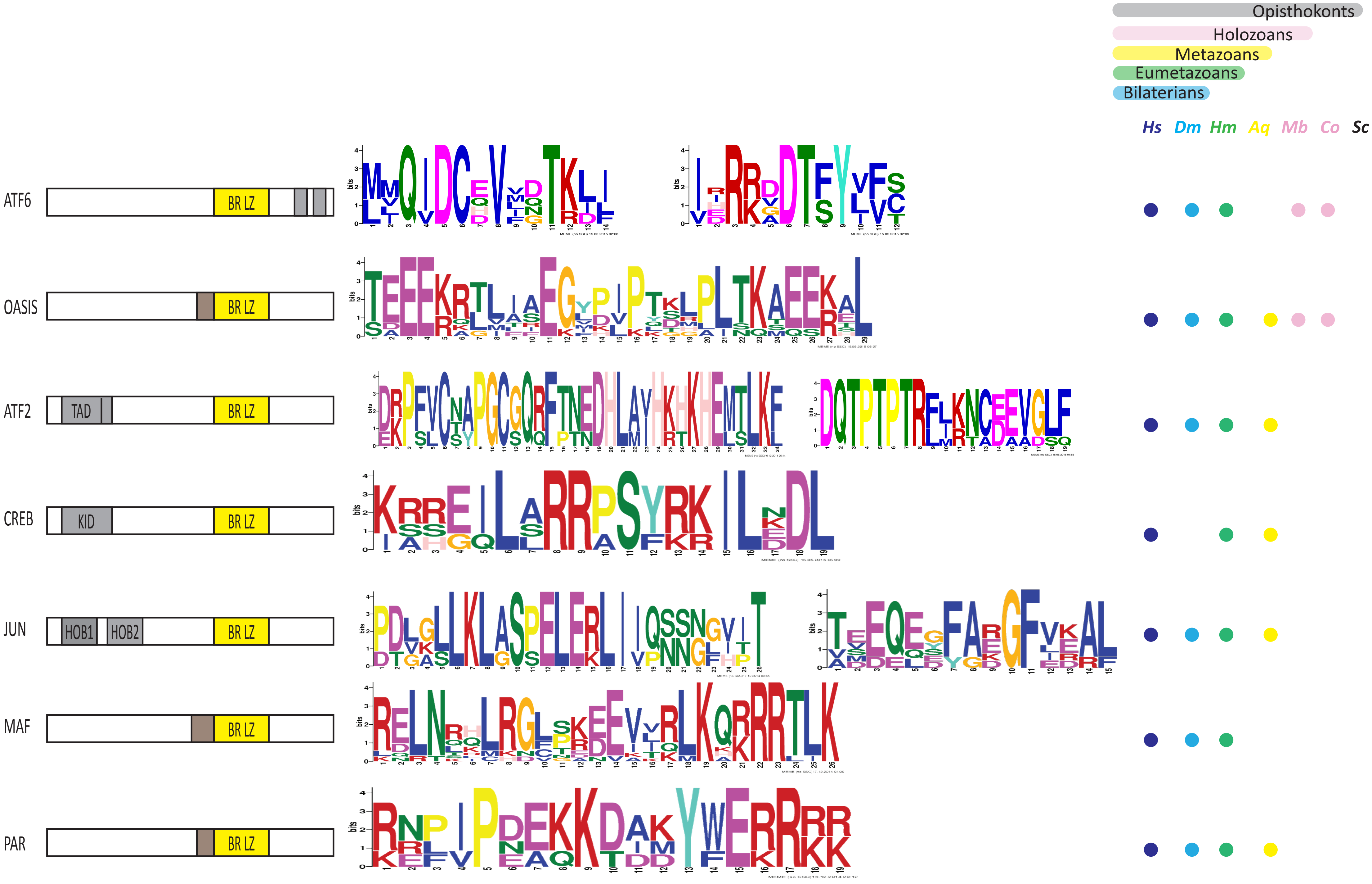

Opisthokonts
Holozoans
Metazoans
Eumetazoans
Bilaterians
Hs Dm Hm Aq Mb Co Sc
ATF6
BR LZ
OASIS
BR LZ
ATF2
TAD
BR LZ
CREB
KID
BR LZ
JUN
HOB1
HOB2
BR LZ
MAF
BR LZ
PAR
BR LZ
bits
